# News and Views

**DOI:** 10.1111/iwj.14024

**Published:** 2022-11-28

## 1 3M

### 1.1 3M V.A.C. Therapy negative pressure wound therapy achieves key medical evidence milestone, surpassing 2000 peer‐reviewed medical journal studies published

3M Health Care's Medical Solutions Division announced that its 3M™ V.A.C.® Therapy negative pressure wound therapy (NPWT) has surpassed a clinical evidence milestone of 2000 published, peer‐reviewed medical journal studies. V.A.C. Therapy is the first and only NPWT solution to garner this number of published studies about its therapy. It is backed by more clinical data than any other brand, accounting for more than 75% of published NPWT clinical evidence.

The clinical studies have been conducted by wound care professionals worldwide and published in journals across the globe, covering a comprehensive range of wound types, wound care settings and study formats, such as case studies, economic studies, randomised controlled trials and more.

“Clinical evidence has always been a foundational element in establishing credibility for V.A.C. Therapy and our NPWT products in the wound care community,” said Ronald Silverman, M.D., 3M Health Care senior vice president of clinical affairs and chief medical officer. “Published studies have also helped to promote adoption of NPWT and spur therapy innovations, including 3M™ Prevena™ Therapy for incision management, 3M™ Veraflo™ Therapy for instillation therapy for open wounds, 3M™ AbThera™ Open Abdomen Negative Pressure Therapy. Our team members in the field are also actively engaged with wound care experts worldwide, working right alongside clinicians to observe the changing nature of wound care and gather feedback about our products, which helps us identify opportunities for innovation.”

Today, V.A.C. Therapy is used across a spectrum of health care settings, from acute care facilities to ambulatory surgical centers, assisted living facilities, and in patients' homes. In the U.S., V.A.C. Therapy is available with 24/7 remote therapy monitoring to support adherence to the therapy. 3M's NPWT portfolio continuously evolves to meet clinician and patient needs. Last year, 3M launched the first‐ever silicone‐acrylic hybrid drape for use with V.A.C. Therapy, the 3M™ Dermatac™ Drape, an innovation designed to be gentle on patients' skin and easy for clinicians to use.

“Today's wound care patients are often sicker and have more comorbidities, making their wounds more complex to treat and increasing the demands on clinicians' time. 3M strives to provide a robust tool selection to address clinicians' unique wound care needs and make our products easier to use to help save their valuable time ‐‐ and ultimately, help transform outcomes and improve lives for wound care patients,” said Dr. Silverman.

## 2 JOHNSON AND JOHNSON

### 2.1 Johnson and Johnson Announces Kenvue as the Name for Planned New Consumer Health Company

Johnson and Johnson took another step forward in establishing two independent, market‐leading companies with the announcement of Kenvue as the name for the planned New Consumer Health Company. The new corporate brand comes to life through a compelling purpose, and a timeless visual brand.

Kenvue will become a standalone leading global consumer health company in 2023, subject to legal requirements including consultation with works councils and employee representatives, as required. Kenvue will continue to touch the lives of over 1 billion consumers worldwide every day through its iconic portfolio of beloved brands such as AVEENO®, BAND‐AID® Brand Adhesive Bandages, LISTERINE®, NEUTROGENA®, TYLENOL®, JOHNSON'S® and will continue its longstanding legacy of innovation.

The Consumer Health segment generated revenue of $14.6 billion in Full‐Year 2021 and, following the planned separation, Kenvue would generate sales in over 100 countries, driven by world‐class innovation capabilities and demonstrated business momentum.

In recent years, Johnson and Johnson has focused the Consumer Health business and advanced its innovation, enabling it to reach more consumers with products that truly make a difference in peoples' lives, while simultaneously expanding margins and delivering healthy financial results. These actions have bolstered positions in Self Care, Skin Health and Essential Health.

Learn more at www.jnj.com. Follow us at @JNJNews.

## 3 MCGILL UNIVERSITY

### 3.1 Drawing inspiration from nature, researchers have developed a medical adhesive that could save lives

Every year around 2 million people die worldwide from haemorrhaging or blood loss. Uncontrolled haemorrhaging accounts for more than 30% of trauma deaths. To stop the bleeding, doctors often apply pressure to the wound and seal the site with medical glue. But what happens when applying pressure is difficult or could make things worse? Or the surface of the wound is too bloody for glue? Drawing inspiration from nature, researchers from McGill University have developed a medical adhesive that could save lives, modelled after structures found in marine animals like mussels and flatworms.

“When applied to the bleeding site, the new adhesive uses suction to absorb blood, clear the surface for adhesion, and bond to the tissue providing a physical seal. The entire application process is quick and pressure‐free, which is suitable for non‐compressible haemorrhage situations, which are often life‐threatening,” says lead author Guangyu Bao, a recently graduated PhD student under the supervision of Professor Jianyu Li of Department of Mechanical Engineering.

In putting the new technology to the test, the researchers found that the adhesive promotes blood coagulation. The adhesive can also be removed without causing re‐bleeding or even left inside the body to be absorbed. “Our material showed much better‐improved safety and bleeding control efficiency than other commercial products. Beyond bleeding control, our material could one day replace wound sutures or deliver drugs to provide therapeutic effects,” says senior author Professor Jianyu Li.

